# Men who have sex with men perceiving that sex with women carries the greatest risk of HIV acquisition: results from a mixed‐methods systematic review in sub‐Saharan Africa

**DOI:** 10.1002/jia2.26402

**Published:** 2024-12-17

**Authors:** Marion Fiorentino, Marie Dos Santos, August Eubanks, Nathan Yanwou, Christian Laurent, Perrine Roux, Bruno Spire

**Affiliations:** ^1^ Aix Marseille Univ, IRD, Inserm, SESSTIM, Sciences Economiques & Sociales de la Santé & Traitement de l'Information Médicale, ISSPAM Marseille France; ^2^ ORS PACA, Observatoire régional de la santé Provence‐Alpes‐Côte d'Azur Marseille France; ^3^ TransVIHMI, Univ Montpellier, IRD, INSERM Montpellier France

**Keywords:** risk perception, key and vulnerable populations, men who have sex with men, women, HIV prevention, Africa

## Abstract

**Introduction:**

In sub‐Saharan Africa (SSA), men who have sex with men (MSM) often have female sexual partners. Their overall risk of acquiring HIV is higher with male partners. Risk perception is associated with HIV knowledge, sexual risk and preventive behaviours. This synthesis aimed to summarize existing data about HIV knowledge and perceived HIV acquisition risk regarding sex with men and with women in MSM in SSA.

**Methods:**

We conducted a systematic literature review of MSM's relationships with women in SSA (PROSPERO‐CRD42021237836). Quantitative and qualitative data related to MSM's perceived risk from sex with men and with women and HIV knowledge (published up to 2021) were selected and synthesized.

**Results:**

Twenty studies were selected. More MSM perceived that the greatest risk of HIV acquisition came from heterosexual/vaginal sex than from homosexual/anal sex (53% vs. 15%; 51% vs. 39%; 42% vs. 8%; 27% vs. 25%; 43% vs. 11%; 23% vs. 13%; 35% vs. 16%, cumulative sample *n* = 4396, six countries). A higher proportion of MSM received preventive information on heterosexual HIV transmission than on homosexual transmission (79% vs. 22%; 94% vs. 67%; 54% vs. 19%; cumulative sample *n* = 1199, four countries). The qualitative synthesis (eight studies) highlighted biology‐ and behaviour‐based misconceptions leading MSM to perceive lower or negligible HIV risk from sex with men, compared to sex with women. These misconceptions were partly fuelled by the predominant focus on heterosexual and vaginal HIV transmission in HIV prevention information.

**Discussion:**

Common misconceptions regarding sexual risk between men remain unaddressed by the heteronormative messaging of HIV prevention. Underestimation by MSM of their HIV acquisition risk with male partners can pose significant barriers to effective HIV preventive behaviours and strengthen the transmission risk from MSM to their female partners.

**Conclusions:**

Improving access of MSM to tailored HIV prevention information and tools that address their practices with male and female partners is crucial. Integrating messages about anal sex into broader public health initiatives, including sexual health programmes targeting the general population, is essential. Further research in diverse settings in SSA is necessary to gain a greater understanding of the drivers and implications of HIV risk perception in MSM.

## 1 INTRODUCTION

Men who have sex with men (MSM) in sub‐Saharan Africa (SSA) frequently engage in sexual and marital relationships with cisgender women (hereafter referred to as women or female partners). This phenomenon is largely driven by heteronormative pressure [1, 2]. HIV strongly affects MSM in SSA, with an estimated incidence of 8 new cases per 100 person‐years in the West and Central Africa region and 5 new cases per 100 person‐years in the Southern and East Africa region. The estimated HIV prevalence among MSM in both regions is 8% and 13%, respectively [3]. Estimated HIV prevalence in women in SSA is lower, at 1% in West and Central Africa and 6% in Southern and Eastern Africa [4]. Compared to insertive vaginal sex, the per‐act risk of HIV acquisition is estimated to be 35 times higher for receptive anal sex and 2.8 times higher for insertive anal sex [5]. HIV acquisition risk for an individual depends on the probability of having an HIV‐positive partner with an unsuppressed viral load, which is influenced by HIV prevalence and antiretroviral treatment (ARV) coverage within the partner's group, and the per‐act risk of acquisition. Consequently, although the individual risk of HIV acquisition is affected by multiple other factors such as frequency of sexual encounters and genital infections [5], MSM most likely have greater exposure to HIV through male partners than through female partners at the population level.

However, high exposure to HIV risk does not necessarily translate into high HIV risk perception among individuals [6, 7, 8, 9]. HIV risk perception is related to multiple factors, such as HIV knowledge and information availability, community influence, cultural background and subjective norms [10] Among the various existing social and structural barriers to access to prevention [11], perceiving oneself at low risk is an important barrier to engaging in preventive behaviours [12, 13, 14]. Therefore, HIV risk perception is a key factor to consider in HIV prevention [15].

In the SSA context, where MSM frequently also have sex with women, understanding how MSM perceive sex with men and with women in terms of HIV transmission risk as well as the factors that might influence these perceptions, is crucial for evaluating their needs regarding HIV preventive information. The objective of the present mixed‐method synthesis was to systematically identify, analyse and summarize existing quantitative and qualitative data on this topic in MSM in SSA.

## 2 METHODS

### 2.1 Systematic review on male bisexuality in SSA

#### 2.1.1 Aim and search strategy

We conducted an extensive systematic review compiling qualitative and quantitative data on male bisexuality in SSA and on MSM sexual or marital relationships with women. The review protocol adhered to the Preferred Reporting Items for Systematic Reviews and Meta‐Analyses (PRISMA) guidelines [16] and was registered prospectively on PROSPERO under number CRD42021237836 [17]. A detailed breakdown of the review procedures, including the literature search strategy, keywords and queries for database search, literature screening, eligibility assessment and data extraction, can be found in our prior articles [1, 2].

Starting from the key concepts “men who have sex with both men and women” (MSMW) and “sub‐Saharan Africa” (SSA), a concept table was created to define keywords suitable for querying databases in both English and French. The search for articles, with no restriction on publication time, was carried out across six databases (PubMed, EMBASE, PsycINFO, Web of Science Core Collection, Google Scholar, CAIRN). All the identified records were exported to the Covidence systematic review software tool (Veritas Health Innovation, Melbourne, Australia).

#### 2.1.2 Selection of studies

Two investigators (NY and MF) independently conducted a two‐step selection process in Covidence, which included (i) screening titles and abstracts and (ii) full‐text screening. Initially, titles and abstracts were screened to exclude articles clearly indicating settings other than SSA or lacking information about MSM. Then, articles were deemed eligible if a full‐text review confirmed they provided relevant information about MSM and women in SSA, regardless of their publication date, inclusion criteria, research design, determinants, implications or topics. Articles identified as duplicates or not in English or French were excluded. Any multiple articles from the same study were merged. The investigators held weekly meetings to address and resolve any discrepancies.

#### 2.1.3 Data extraction

Data from the selected articles were extracted using a custom data extraction form developed in Covidence software, forming the basis of the systematic review database. We created a quality evaluation scale adapted from the QuADS (Quality Assessment for Diverse Studies) tool to assess the methodological quality of the mixed‐method studies included in the review [18, 19]. Data selection and extraction were conducted between 2021 and 2023.

### 2.2 Mixed‐methods synthesis of HIV knowledge and risk perception regarding male and female partners in MSM

Due to the diversity of the extracted data, the authors chose to analyse and present the findings separately by conducting distinct mixed‐method thematic syntheses, each addressing a distinct subtopic on relationships between MSM and women in SSA. The first synthesis provided an overview of MSM sexual behaviours and risks with female partners [1], while the second focused on involvement in steady relationships with women [2].

For this third mixed‐method thematic synthesis, we exclusively selected studies from the systematic review database reporting data about MSM concerning HIV acquisition risk perception in terms of sex with men/women and vaginal/anal sex, and related information received about HIV and HIV knowledge. Both quantitative data (e.g. percentage of a given outcome in an MSM population) and qualitative data (e.g. verbatim from MSM focus groups and interviews) were available. The analysis and synthesis of these data required distinct methods and addressed different sub‐questions (i.e. the extent of perception and knowledge in the MSM population vs. the qualitative insights into the ideas and factors underlying these perceptions and knowledge). Therefore, we employed a “parallel‐results convergent synthesis design,” a method adapted to systematic reviews combining quantitative and qualitative evidence, collected simultaneously but requiring separate analysis and synthesis [20, 21, 22]. It consists of parallel, independent analysis and synthesis of quantitative and qualitative evidence, followed by a common interpretation of the results in the discussion, with the quantitative and qualitative syntheses informing and complementing each other.

#### 2.2.1 Quantitative data synthesis

From cross‐sectional quantitative studies conducted in MSM, we selected percentages of outcomes related to HIV risk perception and knowledge. Due to disparities in the questions asked and the response modalities across the identified studies, there were insufficient data with comparable indicators to pool in a meta‐analysis. Therefore, the data were analysed narratively, that is we grouped the results into different categories and summarized them. The following percentages (relative to the total number of MSM in study samples) were selected:

- **Knowledge of sources of sexual HIV acquisition**:
  - Percentage of MSM respondents who answered “*yes*” to questions such as “*Can you get HIV from vaginal sex?*” (or “*sex with women*” or “*not using a condom during vaginal sex*” depending on the study)
  - Percentage of MSM respondents who answered “*yes*” to questions such as “*Can you get HIV from anal sex?*” (or “*sex with men*,” “*anal sex with men*,” “*anal sex with women*” or “*not using a condom during anal sex*” depending on the study)
- **Perceived riskiest type of sex (for HIV acquisition)**
  - Percentage of MSM respondents who answered the following question or an equivalent: “*What do you consider to be the riskiest type of sex for HIV acquisition?*”: Mutually exclusive response options included *“sex with women,” “sex with men,” “anal sex,” “vaginal sex,'” “oral sex'”* or *“equal risk,”* depending on the study
- **Information received about HIV prevention**
  - Percentage of MSM respondents who answered “*yes*” to the following question or an equivalent: “*Have you received information about HIV prevention related to sex with men?*”
  - Percentage of MSM respondents who answered “*yes*” to the following question or an equivalent: “*Have you received information about HIV prevention related to sex with women?*”

#### 2.2.2 Qualitative data synthesis

Qualitative data on HIV knowledge and risk perception concerning sex with men/women among MSM in SSA, collected from participant interviews and focus group discussions, as well as authors’ comments in selected articles, were imported into NVivo software (version 1.7.1). We utilized a three‐step thematic synthesis methodology developed for qualitative research in systematic reviews [23]. This process involved inductively coding sentences line by line, creating descriptive themes to group initial codes, and then extracting analytical themes through interpretation.

## 3 RESULTS

### 3.1 Included studies and selected data

**Figure 1:**
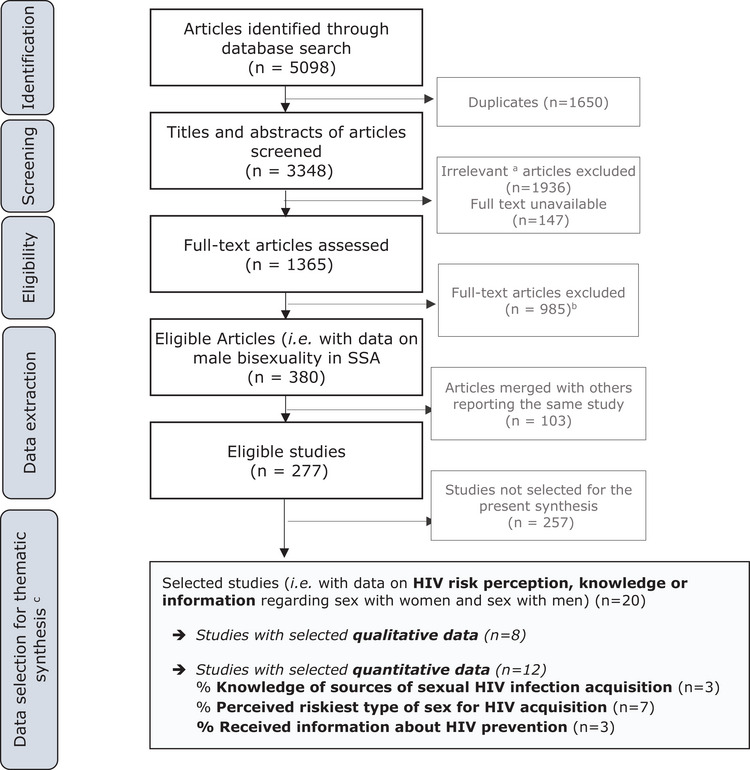
PRISMA Flow chart. (a) That is, the title and abstract indicate no information about MSM, or focuses on settings other than SSA. (b) Reasons to exclude full‐text articles: 364 concerned gay men or MSM in SSA but had no data on MSMW; 180 concerned HIV/sexually transmitted infections epidemics in SSA but did not examine HIV‐bridging from infected MSM to women; 136 did not have enough data (e.g. conference abstract without related published article); 133 concerned a setting other than SSA); 76 investigated men in SSA but had no data on MSMW and male bisexuality; 74 were duplicates; 17 studied women in SSA but had no data on MSMW partners; 3 had publication issues; 2 articles were not in the languages set out in the protocol (i.e. English or French). (c) The first two thematic syntheses, covering different topics, derived from the systematic review have been published separately [1, 2].

For the large‐scale systematic review on male bisexuality in SSA, we initially identified 5098 articles in six literature databases. After automatically removing duplicate entries, we proceeded to screen the titles and abstracts of the remaining 3348 articles. From these, we assessed the full texts of 1365 articles, and identified 277 studies from which we extracted various types of data related to male bisexuality in SSA, that constituted the systematic review database (Figure 1). For the present synthesis, of these 277 studies, we selected all the studies that contained quantitative or qualitative data about HIV knowledge, information or perceived risk regarding sex with men/women in MSM, resulting in a final selection of 20 studies.

### 3.2 Quantitative synthesis

Twelve studies supplied quantitative data, collected in Botswana, Burkina Faso, Cote d'Ivoire, Kenya, Lesotho, Malawi, Namibia, Swaziland, Uganda, and in a 22‐SSA country online study, involving a total of 5281 MSM (published between 2011 and 2020).

#### 3.2.1 Knowledge of sources of sexual HIV acquisition

**Figure 2:**
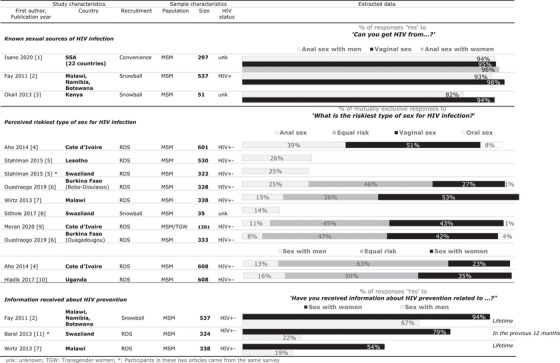
Synthesis of studies with quantitative data about HIV knowledge, HIV acquisition risk perception and HIV information received in men who have sex with men (MSM) in SSA in terms of sex with men and with women.

In each of the three studies assessing knowledge of the sexual sources of HIV acquisition (cumulative sample *n* = 885 MSM) [24, 25, 26], a high percentage of MSM were aware that anal sex and sex with men can transmit HIV (Figure 2). These values were slightly lower than the percentages who knew that vaginal sex and sex with women are sources of HIV acquisition: 94% (for anal sex with men) versus 95% (for vaginal sex) and 96% (for anal sex with women); 93% (for anal sex with men) versus 98% (for vaginal sex); 82% (for anal sex with men) versus 94% (for vaginal sex).

#### 3.2.2 Perceived riskiest type of sex for HIV acquisition

Seven studies evaluated MSM perceptions of the riskiest type of sex for HIV acquisition and reported the proportions of mutually exclusive responses (cumulative sample *n* = 4396 MSM) [27, 28, 29, 30, 31, 32, 33]. In all seven, 1.1 to 5.3 times more MSM designated vaginal sex or sex with women as the riskiest type of sex than those who designated anal sex or sex with men (53% vs. 15%; 51% vs. 39%; 42% vs. 8%; 27% vs. 25%; 43% vs. 11%; 23% vs. 13%; 35% vs. 16%, Figure 2). The proportion of MSM responding “equal risk” (reply allowed in six studies) varied between 36% and 63%. A relatively small percentage of MSM perceived oral sex to be the riskiest type of sex (1−8%, reply allowed in four studies). Besides these seven studies, two others only reported the proportions of MSM replying “anal sex” as the riskiest type of sex, which were 25% and 26%.

#### 3.2.3 Received information about HIV prevention

In three studies studying HIV prevention information provided to MSM (cumulative sample *n* = 1199 MSM) [25, 27, 34], 1.4 to 3.6 times more MSM received information about sex with women than those who received information about sex with men: 94% versus 67%; 79% versus 22%; 54% versus 19% (lifetime or in the previous 12 months, depending on the study, Figure 2).

### 3.3 Qualitative synthesis

Among the 20 selected studies, eight studies collected qualitative data in Ethiopia, Kenya, Namibia, South Africa, Tanzania and Zimbabwe (published between 2002 and 2019) [35, 36, 37, 38, 39, 40, 41, 42].

#### 3.3.1 Perception that the risk of HIV acquisition from sex with men is negligible or low compared to sex with women

The main theme that was identified from the qualitative synthesis (Table 1) was the misconception by a proportion of MSM participants in all eight included studies that sexual intercourse with men carries a negligible or lower risk of HIV acquisition compared to sex with women. This was intertwined with various other misconceptions that women are at greater biological or behavioural risk of transmitting HIV than men. Frequent examples were the beliefs HIV is transmitted only through fluids which are present in the vagina but absent in the anus, and that transmission only occurs through bleeding. In some cases, these particular misconceptions were fuelled by misunderstandings of biology or of HIV information received in schools. For example, some MSM believed that because the vagina was “more connected to blood” and “had more membranes” (compared to the anus), the risk of HIV transmission was higher. Others believed that going to the toilet to “defecate” semen or penile/anal washing following anal sex prevented HIV acquisition; this contributed to the perception of a low risk of HIV acquisition from sex with men. MSM also cited behavioural beliefs that MSM have better hygiene, have fewer choices of male partners, engage in intercourse less frequently, and that in comparison to MSM, women are more frequently sexually solicited by men and, therefore, are more likely to have multiple partners, translating into a higher risk of acquiring HIV than MSM. MSM cited the small size of the MSM population compared to the heterosexual population, and the belief that most MSM do not have sex with women, as reasons to justify their perception that the risk of HIV transmission within the MSM population is low.

**Table 1:** Synthesis of studies with qualitative data about HIV information, knowledge and risk perception regarding sex with men and with women in men who have sex with men (MSM) in SSA

*Characteristics of qualitative studies*				
*First author, publication year*	*Country*	*Recruitment*	*Population*	*Sample size*
Tsang 2019 [40]	Zimbabwe	Convenience	MSM sex workers	15
Gebreysus 2009 [39]	Ethiopia	Snowball	MSM	40
Lorway 2006 [42]	Namibia	Convenience	MSM	31
Okal 2009 [38]	Kenya	Convenience	MSM sex workers	36
Onyango‐Ouma 2005 [35]	Kenya	Snowball	MSM	57
Tadele 2010 [37]	Ethiopia	Snowball	MSM	30
Lockhart 2002 [36]	Tanzania	Snowball	Street boys having male and female partners	75
Lane 2008 [41]	South Africa	Convenience	MSM	32

***Qualitative data synthesis***	
*Themes*	*Verbatim excerpts from the selected studies (with characteristics of interviewees, where available)*
*Perception “* ***The risk of HIV acquisition from sex with men is negligible or low compared to sex with women”***	
HIV is only transmitted from women, not from men	“Seven of [the male sex workers] **believed that HIV was only transmitted through sexual intercourse with women but not with men**: *‘I know that if you sleep with a woman without using a condom you can get HIV, but* ***I don't know about sleeping with another man and whether you can get the virus’***.*”* (author's citation and interviewee discourse, Zimbabwe [40]) “Almost all [participants] have misconceptions, as **they consider homosexual acts to be protective** compared with heterosexual acts*.”* (author's citation, Ethiopia [39]) “*Most of the men I know who have girlfriends are saying that they prefer to have sex with us moffies* [gay men] *because* ***they don't want to catch STDs cheating on them, or HIV*** *, or pregnancy. Most of them think* ***they can even have sex with men without a condom because they think it is less risky than sex with a woman***.” (interviewee discourse [20 y], Namibia [42]) *“There is AIDS and all these but* ***I have never heard of anybody having a sexually transmitted infection in the anus*** *…* ***Mostly I think this disease [AIDS] is found in the vagina and mouth*** *, but I still can't understand it well.”* (interviewee discourse [24 y, MSM with female partners], Kenya [38])
***Beliefs underlying misconceptions***	
*About biology*	
HIV transmits through vaginal lubrication/fluids Anal sex does not transmit HIV because the anus is dry The vagina is “connected to blood” The vagina has “lots of membranes” The expulsion of semen from the anus by defecating prevents HIV acquisition	*“*Some MSM believe incorrectly that HIV is not transmitted via anal intercourse, and is transmitted only through women. These MSM typically believed that vaginal sex was the riskiest type of sex **because of the amount of fluids involved as compared to anal sex**: ‘*Vaginal sex is like swimming in a pool of* ***water [fluids], which increases the risk of HIV transmission*** *’.”* (authors’ citation and interviewee discourse [29 y, unemployed], Kenya [35]) “*I think sex between a man and a woman is more risky and exposes people more to HIV*. ***The main source of the virus is mucus or a liquid lubricant in the vagina*** *, which* ***you don't find in anal cavities*** *and therefore, it is safer than vaginal sex.”* (interviewee discourse, sex worker, Ethiopia [37]) “*I believe that you can't get HIV from having sex with a* [gay man] *because* ***there is no mixing of fluid there [he points to the anal region]; it is dry***.*”* (interviewee discourse [24 y, MSM with female partners], Namibia [42]) *“I see it [having sex] as more risky with a woman* ***because there is blood connected*** *. In a man I do not know where the blood is connected. In a woman you are directly connected … You can tell* ***inside they are wetter than a man*** *.”* (interviewee discourse, MSM with female partners), Namibia [42]) “***I think it is more risky in heterosexual sex*** *… because* ***in biology in school*** *we would have the diagram and the anatomy [of a vagina]. You could see that all the way inside [a woman]* ***it is wet*** *. It is like* ***full of membranes*** *. But* ***men don't have as many membranes*** *[in their anus].”* (interviewee discourse [19 y, gay], Namibia [42]) *“How can a gay man get AIDS?* ***You won't get AIDS from a man because when you go to the toilet, you kick out the sperm, it goes out the body*** *.”* (interviewee discourse [19 y, gay], Namibia [42])
*About behaviours*	
Sex between men is not considered real sex	“**None of the boys believed AIDS could be transmitted through behaviors related to** kunyenga [**male‐male sex**] because **they did not consider it ‘real sex’**. They felt strongly that AIDS could only come from females*.”* (authors’ citation, Tanzania [36])
	
Sex between men is not as frequent as heterosexual sex	“*I think sex with a woman transmits HIV more [than sex between to men]…* ***When a man and a woman have sex, the chances of having blisters and sores are higher*** *, as they are* ***likely to have sex nonstop all night*** *. But such people [gays] have sex once or twice.”* (interviewee discourse, sex worker, Ethiopia [37])
Women are more likely to have risky behaviours (multiple partners, no condom use) than MSM	*“A* ***woman is more dangerous*** *because* ***other men have their eye on her*** *, and* ***she will run off with other men*** *. The* [gay men] *make me wear condoms sometimes … but some of my straight friends don't wear them. It is* ***better to have sex with a* [gay man] *for a one‐night stand*** *.”* (interviewee discourse [21 y, MSM having female partners], Namibia [42]) *“With a woman* ***you are attracted to their shape, their curves, but so are other men*** *. She may run off with other men. But* [gay men] *are safer because* ***they do not sleep with women and they will be faithful to you*** *.”* (interviewee discourse [24 y, MSM having female partners], Namibia [42])
MSM have good hygiene, preventing HIV	*“From what I know, I think the contact there [in* ***sex with women] is a lot more risky*** *. I don't think it is transmitted that much through unprotected male‐to‐male sex. (…)* ***Some guys usually wash up immediately after sex*** *, they clean themselves with lemon and the like*. ***And if there is no bleeding, should there be any concern*** *?”* (interviewee discourse [sex worker], Ethiopia [37])
The small MSM population size decreases the probability of male‐male sex	*“There aren't that many* [gay men], *they are rare. There is just a few of us and we have sex with each other inside that small group. And since most gays don't go with women, the sex is pretty limited, because the possibilities are limited. So that reduces the risks of getting HIV.”* (interviewee discourse [sex worker], Ethiopia [37])
***Structural context***	
No/insufficient information about male‐male sex and anal sex provided by HIV prevention programmes, media and schools	“In Kenya, the **silence of HIV prevention programmes and lack of media reports on anal sex** may reinforce perceptions that anal sex is not a high‐risk sexual practice. Some informants held a conviction that they would not acquire HIV, despite having unprotected anal sex with both male and female partners. Some participants believed anal sex was ‘safer’ than vaginal sex and were more likely to report condom use for vaginal sex*: ‘I don't believe that most men use condoms* ***because with the information I get illness is in the vagina and not the anus…I have never heard of anybody getting illness from the anus, even from our teachers*** *’.”* (author's citation and interviewee discourse, Kenya [38]) *“I heard that you are supposed to wear a condom between men but* ***I don't know how you can get HIV from a man***. ***I didn't learn about that in school*** *. It is in the blood cells. It is different down there* [points to anus]*. It is not like a woman where there is blood and body fluids.”* (interviewee discourse [17 y, gay], Namibia [42]) “**Through continual exposure to HIV/AIDS awareness campaigns** in Namibia, most informants thought of AIDS as a disease of ‘dangerous bodily fluids’: breast milk, vaginal fluid, blood and semen. Many informants not only viewed **female bodies as wetter**—because they lactated, menstruated, and had many ‘membranes’—but also considered them **more contagious and polluted than male bodies**. (…) The [gay man] was conceived of **as the purer substitute for the polluted female body**.” (authors’ citation, Namibia [42])
Invisibility of MSM in healthcare services	“In elaborating health‐seeking strategies that promoted their invisibility, non‐gay identified (NGI) MSM asserted a right to privacy in healthcare that was an alternative interpretation of their constitutional guarantee of quality. But **protecting privacy through invisibility also prevents many NGI MSM from seeking sexual health information** and services from sympathetic public sector HCW or from LGBT CBO. For some, this could have the unintended consequence of reinforcing erroneous beliefs about HIV or STI transmission. For example, one straight‐identified participant seemed certain that his STI could only be acquired heterosexually*: ‘I don't [disclose my same‐sex behavior] … because when I go to the doctor* ***it is not for male sexual contact that I have contracted an STD’*** *.”* (author's citation and interviewee discourse [straight‐identified MSM], South Africa [41])
***Psychosocial outcomes***	
Attempt to decrease stigma towards MSM “Boundaries of sexual safety”	“***I don't believe zegoch [gay men] can get HIV*** *. I once almost got into a fight with one of my [straight] friends*. ***I was telling him that I appreciate such [gay] people. And he was about to give me a knocking*** *, and was asking me what on earth made me say that, he was a macho type you know, quick to get into a fight and all. And I told him I appreciate them* ***because they can't get HIV*** *, since they only go out with guys and therefore can't possibly get AIDS. That's what I believe.”* (interviewee discourse [sex worker], Ethiopia [37]) “All of these point to the fact that **male‐to‐male sex and the homosexual community are perceived as AIDS free**. Compared with studies conducted in Western society, heterosexual young men perceive ‘heterosexual community’ or heterosexual sex per se as safe. Flood (2003, p. 363) coined such classification as ‘**boundaries of sexual safety’**. Likewise, throughout human history, many epidemic diseases have often been blamed on others, on outsiders. Similarly, **AIDS has been considered the problem of ‘others’ all along in its history**, and it has been a metaphor for human differentiation by race, class, sexual identity, and gender.” (authors’ citation, Ethiopia [37])
*Perception* “***There is a high risk of HIV acquisition from sex with men”***	
***Related knowledge about HIV***	
A lack of lubrication increases the risk of HIV transmission	*“I think you are more likely to get HIV from having sex with other men because* ***women have natural lubricants that they excrete during intercourse*** *, and their* ***vagina can stretch quite well too*** *. But when you are having sex with men, it isn't like that. I mean we use Vaseline but that is just about it. And at times all you have for lubrication is just your saliva; you know if it is tight you put some saliva on your penis [nervous laugh].”* (interviewee discourse [key‐informant], Ethiopia [37])
Multiple partners and bleeding during anal sex increase the risk of HIV transmission between men	*“I don't know that much about the science of HIV transmission but gays rarely use condoms, and they have sex with* ***many partners*** *, you rarely see them being faithful to one sexual partner, and even the type of sex is different and more exposing to HIV infection, like oral sex, sucking and so on. And there are some guys with huge penises who might* ***bleed their partners during anal sex*** *. And* ***since they do not use condoms, I think that opens more doors for HIV transmission*** *.”* (interviewee discourse [key‐informant], Ethiopia [37])

The authors of qualitative studies pointed out two structural factors contributing to these various misconceptions in SSA countries where the studies were conducted. The first is the predominant focus on heterosexual and vaginal HIV transmission in HIV prevention programmes aimed at the general population both through the media and through schools with insufficient or no information about anal sex or sex between men. This led some MSM to incorrectly perceive that sex with men carried a negligible or lower HIV risk compared to sex with women. The second structural factor is that due to confidentiality and privacy issues related to stigma in healthcare facilities, MSM do not discuss their same‐sex behaviours with healthcare workers, preventing them from receiving prevention information about male‐male sex.

One study [37] referred to the “boundaries of sexual safety,” citing Flood [43], to discuss the perceptions of MSM that their community is HIV‐free. This term refers to the long‐held belief in many communities that AIDS is a problem for “others” (i.e. other communities). The perception of belonging to an HIV‐free community allowed some MSM to reclaim a sense of worth for themselves and for the MSM community in response to the persistent stigma they face.

#### 3.3.2 Perception that there is a high risk of HIV acquisition from sex with men

The perception that sexual intercourse with men carries a high risk of HIV acquisition was reported by only two “key‐informant” MSM in one qualitative study (Table 1). These informants justified their answers by citing that multiple partners, a lack of lubrication and bleeding during anal sex all increase the risk of HIV transmission in male‐male sexual encounters. This suggests that active involvement in the MSM community facilitates the understanding and adoption of accurate information about HIV transmission.

## 4 DISCUSSION

The current quantitative synthesis suggests that while a high proportion of sub‐Saharan African MSM included in the studies were aware that anal sex poses a risk of HIV acquisition, anal sex or sex with men was not perceived as carrying the highest risk of HIV transmission. Instead, they frequently perceived that vaginal sex carried an equal or higher risk. This finding contrasts with research in Europe where MSM perceived the risk of HIV acquisition with male sexual partners to be much higher than with their female partners, partly because they assumed—in direct contrast with our qualitative finding—that women were less likely to have multiple partners. This led them to less frequently use condoms with female partners than with male partners [44].

Our qualitative synthesis reflected the quantitative synthesis finding about the misconception regarding the higher risk of vaginal sex. It highlighted several beliefs which foster this misconception, including the common inaccurate belief that natural vaginal lubrication carries diseases, and that anal sex is, therefore, safer because of the absence of fluids. This perception is possibly rooted in prevalent beliefs in the general populations of men and women in several SSA countries where vaginal lubrication is associated with moral consideration (lubrication being perceived as a sign of non‐virginity, infidelity, promiscuity or prostitution), dirtiness and something that carries disease, and that the “normal” state of a vagina—including for men's sexual pleasure—is dry [45, 46]. This perception of vaginal fluids prompts many women to perform vaginal drying practices, which increase the risk of HIV transmission [47, 48].

Other misconceptions revealed in our qualitative synthesis regarding HIV in MSM, included the belief that post‐sex genital washing prevents HIV acquisition (the ineffectiveness of this action has been demonstrated [49]), and that women are behaviourally at higher risk to be HIV positive, because heterosexual men and women are perceived as more sexually active and having more partners, compared to MSM. Our synthesis suggests that misconceptions and lack of knowledge impact the perception of different components of HIV risk, as MSM perceive the HIV risk associated with sex between men as low because they view both the per‐act transmission risk and the probability of encountering an HIV‐positive partner as lower with male partners compared to female partners. Previous research in South Africa and Namibia identified other misconceptions which possibly act as barriers to MSM implementing HIV prevention behaviours, including HIV‐related conspiracy beliefs [50], beliefs that HIV can be transmitted through witchcraft and supernatural means, sharing food and mosquito bites, and the belief that a seemingly healthy person cannot be living with HIV [51].

The qualitative synthesis underscored the focus on heterosexual transmission information and the insufficient or absent information about anal sex in HIV prevention messages as factors leading MSM to perceive anal sex as “not real sex,” “less dirty” than vaginal sex or not associated with any risk of HIV transmission because of the absence of fluids. It is important to point out that in several countries, the absence of information about anal sex in HIV prevention programmes contributes to the perception in the general population that anal sex is safe. This misconception can encourage anal sex over vaginal sex in heterosexual encounters [52]. The omission of sex between men in prevention messages and the lack of adequate MSM‐targeted HIV prevention services are additional structural factors contributing to misconceptions among MSM about the risk of HIV. Our quantitative findings showed that only a small proportion of MSM had received HIV prevention information regarding sex with men. This reflects UNAIDS data, which found that out of the 11 countries in SSA for which data were available, less than 50% of the MSM population were reached by HIV prevention programmes in five of those countries [53]. Moreover, this lack of prevention in MSM is reflected in a recent systematic review on HIV prevention interventions in SSA which only identified interventions focusing on heterosexual transmission and vertical transmission [54].

Our findings have several possible implications in terms of the risk of HIV transmission between MSM and between MSM and women in SSA. First, we can hypothesize that the frequent misconception in our synthesis that sex with men carries a negligible or lower risk compared to sex with women, is a major barrier to MSM willingness and ability to engage in preventive behaviours with their male partners. Even if the pathway between behaviours, risk perception and knowledge is complex, and having high HIV knowledge and self‐perceiving at high risk do not necessarily lead to the adoption of preventive behaviours, HIV risk perception plays an important role in driving preventive behaviours [6, 7, 8, 9]. In particular, MSM who perceive themselves to be at low risk or no risk are less likely to adopt preventive behaviours, such as condoms or pre‐exposure prophylaxis (PrEP), with male partners [12, 13, 14]. In men who only have sex with men (i.e. exclusive MSM), it may foster a sense of safety regarding HIV risk. Compared to MSMW, they more frequently had no or low HIV risk perception, in a study in Mozambique [55].

Second, the common perception in MSM that sex with women carries a greater risk of HIV acquisition compared to sex with men, may not automatically translate into a strong likelihood that they will adopt preventive behaviours with their female partners. Previous mixed‐methods findings from the same systematic review we used here, showed that MSM frequently engage in sexual and marital relationships with women in SSA and that there are significant barriers to HIV testing, counselling and condom use with these female partners, including the desire to have children and the need to keep their MSM behaviours secret [1, 2]. Indeed, the process of deciding whether or not to adopt HIV prevention behaviours goes far beyond responding solely to perceived biological risk; it also involves psychosocial factors, relational dynamics and economic considerations, along with potential associated costs [56]. Additionally, the female partners of MSM may not be very willing or able to negotiate condom use with their MSM partners either because they are unaware of being exposed to the risk through their partner [2], or because of prevailing gender inequities and power imbalances within relationships [57, 58]. Compared to other women, female partners of MSM are probably at higher risk of HIV acquisition, as HIV prevalence among SSA MSM is generally higher than in the general population of adult men [11]. It is important to point out that all the studies we reviewed exclusively examined HIV risk knowledge and perceptions concerning HIV acquisition. No study investigated these dimensions in terms of transmission *from* MSM participants *to* their male or female partners, or indirectly to their children through vertical transmission.

A lack of information was one of the drivers behind MSM misconceptions in terms of the risk of HIV from sex with men and women. This may impede HIV preventive behaviours with male partners and hinder the initial stage of the HIV cascade of care (condoms, PrEP and HIV/STI testing) while not necessarily increasing preventive behaviours with female partners. This gap in accurate HIV knowledge adds to the already numerous barriers encountered by MSM in SSA at the different steps of the HIV care cascade (access to condoms and PrEP, HIV/sexually transmitted infections (STI) testing and treatment, retention in care and viral suppression) [11, 59, 60, 61], and may contribute to the risk of HIV bridging from MSM to women.

This mixed‐method synthesis has inherent limitations. First, data were only available for a small number of SSA countries, with disparities across different regions in SSA (no qualitative data were available for West Africa), and in the available indicators provided by the quantitative studies; this prevented us from performing a robust quantitative synthesis through a formal meta‐analysis.

Second, it was impractical to comprehensively contextualize and generalize all the findings, given the vast diversity within the sub‐Saharan African region in terms of culture, political contexts regarding same‐sex behaviours, health systems and the HIV epidemic. Third, as MSM are a particular hidden population, the data presented in the included studies are not representative of the entire MSM population of SSA countries. Having said that, the majority of quantitative studies included used snowball‐ or respondent‐driven sampling, which are recognized as more efficient for recruiting hard‐to‐reach populations than convenience sampling. Respondent‐driven sampling is also designed to mitigate sampling biases [62, 63]. Only two quantitative studies used convenience sampling. Fourth, the included quantitative studies did not directly measure self‐risk appraisal of MSM with their own male and female partners but rather their HIV risk perception regarding anal/vaginal sex and sex with men/women in general. Moreover, they did not distinguish receptive from insertive anal sex in questions about the perceived riskiest type of sex (i.e. in comparison to vaginal sex). Finally, the synthesis highlighted a lack of recent qualitative studies from several different countries. Specifically, most included studies were published in 2010 or earlier, and most insights were provided by studies from Namibia and Ethiopia.

In terms of strengths, to the best of our knowledge, this is the first mixed‐method systematic synthesis of HIV information and HIV acquisition risk perception in MSM in SSA in terms of sexual intercourse with male and female partners. It revealed specific avenues for research and public health consideration, including the possibility that the perceived HIV risk among MSM may be frequently inversely related to their real exposure to risk, given various socio‐cultural factors. Their underestimation of the risk may increase the likelihood of HIV acquisition from male partners and subsequent transmission to female partners.

## 5 CONCLUSIONS

Our synthesis highlighted the need for more comparable and comprehensive data about HIV risk perception in MSM in SSA. Further quantitative and qualitative data are needed to explore the drivers of HIV risk perception in its different components (perceived per‐act risk transmission and perceived probability to have an HIV‐positive partner in a given group) and its behavioural implications on sexual choices, relationships, and the adoption of preventive measures with male and female partners among MSM in SSA. Future research should consider individual risk appraisal [64], and take into account specific cultural, social and economic contexts within the SSA region, such as the impact of gender and sexuality norms and stereotypes. It should also consider the coverage, targeting and content of HIV prevention programmes.

The legal status of same‐sex behaviours differs substantially across sub‐Saharan African countries, from non‐criminalization (e.g. Botswana, Burkina Faso, Cote d'Ivoire, Lesotho) to prison sentences < 8 years (e.g. Ethiopia, Swaziland, Zimbabwe), ≥14 years (e.g. Kenya, Malawi), and even life imprisonment or the death penalty (e.g. Tanzania, Uganda). Our findings suggest that MSM are marginalized in HIV prevention, including in countries where same‐sex behaviours are not criminalized. This may reflect a broader social context hostile to MSM, which is not necessarily tied to the legal status of these behaviours.

Strengthening MSM community organizations and informants and tailoring HIV prevention programmes specifically for MSM is crucial for developing effective preventive measures. This includes dispelling misconceptions about HIV and raising awareness of HIV transmission risks in its various components such as per‐act transmission risk and HIV prevalence within groups, both within male‐male partnerships and interactions with female partners. Implementing information campaigns targeting MSM should address horizontal transmission and acquisition risk associated with anal and vaginal sex, as well as the potential for indirect transmission to their children through vertical transmission by female partners of MSM. Such campaigns could be deployed across diverse MSM community settings, including social networks, venues and dating apps. Moreover, it is essential to provide individual counselling that acknowledges the possibility of concurrent relationships with both male and female partners, and includes discussions about HIV knowledge and personal appraisal of the risks of acquisition and transmission. Information about HIV prevalence and incidence among MSM should be conveyed during these individualized sessions, or in private MSM group discussions, rather than in public campaigns, to minimize the risk of stigmatizing MSM. Given the barriers to condom use with female partners, community‐based support for MSM should offer various HIV testing tools and PrEP regimens adapted to individual circumstances and relationships with male and female partners. Moreover, a previous systematic review highlighted the importance of integrating positive symbolic meanings such as sexual pleasure, love, responsibility and trust, in order to enhance acceptability and promote adherence to biomedical HIV prevention interventions [56].

Reaching hidden MSM in MSM community‐based settings in SSA is extremely challenging, as issues related to stigma, discrimination and criminalization are involved [65]. To address common HIV‐related misconceptions—both in the general population and in MSM not reachable through community networks—it is crucial to incorporate explicit messages about anal sex within broader public health initiatives such as HIV prevention and sexual health promotion programmes aimed at the general population. Furthermore, health facilities within the global public and private health systems must be strengthened to empower healthcare workers to effectively reduce MSM discrimination and stigma, and to deliver appropriate HIV prevention and care that is tailored to patients’ behaviours and needs, including those of MSM. More generally, in the interest of public health, the involvement of policymakers and community leaders is necessary to mitigate this unfavourable socio‐political context and the multiple vulnerabilities of MSM.

## COMPETING INTERESTS

The authors have no competing interests to declare.

## AUTHORS’ CONTRIBUTIONS

The steering committee, which comprised AE, PR, CL and BS, oversaw the systematic review process and actively participated in the design and interpretation of the results. MF developed the protocol, conducted the literature search, screened and assessed articles, extracted and managed data, performed data analysis and synthesis, and drafted the manuscript. MDS participated in the qualitative data analysis. NY participated in the screening and assessment of articles, as well as data extraction. All authors collaborated on revising the manuscript, and all approved the final version.

## Data Availability

Data sharing is not applicable to this article as no new data were created in this study.
